# Association between functional genetic variants in retinoid X receptor*-α/γ* and the risk of gestational diabetes mellitus in a southern Chinese population

**DOI:** 10.1042/BSR20211338

**Published:** 2021-10-19

**Authors:** Xiang-yuan Yu, Li-ping Song, Hui-ting Zheng, Shu-dan Wei, Xiao-lan Wen, Bo Huang, Da-bin Liu

**Affiliations:** 1 Institute of Preventive Medicine, School of Public Health, Guilin Medical University, Guilin 541100, China; 2 Department of Epidemiology and Health Statistics, Guilin Medical University, Guilin 541100, China; 3 Fujian Key Laboratory of Women and Children’s Critical Diseases Research, Fujian Maternity and Child Health Hospital, Fuzhou 350001, China

**Keywords:** association study, gestational diabetes mellitus, retinoid X receptor, single nucleotide polymorphisms, susceptibility

## Abstract

To clarify the effect of *retinoid X receptor-α/γ* (*RXR-α/γ*) genes functional genetic variants (*RXR-α* rs4842194 G>A, *RXR-γ* rs100537 A>G and rs2134095 T>C) on the risk of gestational diabetes mellitus (GDM), a case–control study with 573 GDM patients and 740 pregnant women with normal glucose tolerance was performed in Guangxi area of China. An odds ratio (OR) with its corresponding 95% confidence interval (CI) was used to assess the strengths of the association between genetic variation and GDM. After adjustment of age and pre-BMI, the logistic regression analysis showed that the rs2134095 was significantly associated with GDM risk (CC vs. TT/TC: adjusted OR = 0.71, 95% CI = 0.56–0.90) in all subjects, and this result remained highly significant after Bonferroni’s correction for multiple testing (*P*=0.004). The stratified analysis showed that rs2134095 was significantly associated with the risk of GDM among age > 30 years (adjusted OR = 0.61, 95% CI = 0.39–0.97), BMI > 22 kg/m^2^ (adjusted OR = 0.46, 95% CI = 0.30–0.70), systolic blood pressure (SBP) > 120 mmHg (adjusted OR = 1.96, 95% CI = 1.14–3.36), glycosylated hemoglobin A1c (HbA1c) < 6.5% (adjusted OR = 1.41, 95% CI = 1.11–1.78), TG ≤ 1.7 mmol/l (adjusted OR = 2.57, 95% CI = 1.45–4.53), TC ≤ 5.18 mmol/l (adjusted OR = 1.58, 95% CI = 1.13–2.22), high-density lipoprotein cholesterol (HDL-c) ≤ 1.5 mmol/l (adjusted OR = 1.70, 95% CI = 1.16–2.49) and low-density lipoprotein cholesterol (LDL-c) > 3.12 mmol/l (adjusted OR = 1.47, 95% CI = 1.08–2.00) subjects, under the recessive genetic model. We also found that rs2134095 interacted with age (*P_interaction_*=0.039), pre-BMI (*P_interaction_*=0.040) and TG (*P_interaction_*=0.025) influencing individual’s genetic susceptibility to GDM. The rs2134095 T>C is significantly associated with the risk of GDM by effect of a single locus and/or complex joint gene–gene and gene–environment interactions. Larger sample-size and different population studies are required to confirm the findings.

## Introduction

Gestational diabetes mellitus (GDM) is defined as different degrees of impaired glucose tolerance that is first recognized during pregnancy, often manifested as insufficient islet secretion and insulin resistance, which seriously threatens maternal and child health [1]. Worldwide, it affects approximately 2–20% of all pregnancies [2]. Clinical and epidemiological studies have demonstrated that GDM is associated with maternal and perinatal complications, such as fetal macrosomia, pre-eclampsia, preterm birth, spontaneous abortion, respiratory distress syndrome, small for gestational age (SGA), large for gestational age (LGA), shoulder dystocia, hypoglcemia, polycythemia etc [3–5]. As yet, the main risk factors are known as older age at pregnancy, obesity, family history of type 2 diabetes (T2DM) and past history of GDM, previous poor obstetric history and genetic factors [6–10]. So far, the pathogenesis of GDM is not clear.

GDM was thought to have similar pathophysiological mechanisms with T2DM, such as insulin resistance, β-cell dysfunction and defects in insulin action [11]. Retinoic acid, as a product of retinol catabolism, can maintain the activity of vitamin A by activating retinoic acid receptor (RAR) and retinoid acid X receptor (RXR). Studies have confirmed that retinoid is highly associated with diabetes, islet function, and glucose and lipid metabolism by regulating the transcription of downstream target genes [12–14]. The RXRs are nuclear receptors that mediate the biological effects of retinoids by their involvement in retinoic acid-mediated genes activation. It functions as transcription factors by binding as heterodimers to the specific sequences in the promoter of target genes. In addition, RXRs are members of the vitamin D (VD) pathway that regulate the transcription of vitamin D receptor (VDR) and helps to repair islet β cells, improve insulin resistance and promote the glucose and lipid metabolism [15–18]. It is reasonable to believe that RXRs play a key role in the pathogenesis of GDM.

Single nucleotide polymorphism (SNP) mainly refer to the polymorphism of DNA sequence caused by the conversion or transversion of a single nucleotide, as the most common genetic variation in humans, accounting for more than 90% of all known variants. It has been confirmed that some variants located in the gene may directly affect the protein structure or expression level, and may represent some factors in the genetic mechanism of GDM [19]. A genome-wide association study (GWAS) from South Korea showed that T2DM-associated genetic variants were also associated with GDM risk, such as *SLC30A8* rs3802177, *CDKAL1* rs10440833, *CDKN2A/2B* rs10965250 etc [20]. In addition, studies have shown that the genetic variation rs10830963 of *MTNR1B* gene is associated with the pathogenesis of T2DM by affecting the expression of MTNR1B [21], and carrying the *MTNR1B* rs10830963G allele significantly increased the odds of antenatal insulin therapy (AIT) in pregnant women of GDM with pre-pregnancy BMI > 29 kg/m^2^ [22]. The above mentioned evidence revealed that inherited genetic factors are also contributed to the genesis and development of GDM.

The chromosomal 1q21-24 is identified as the susceptible region of T2DM, and the *retinoid X receptor-γ* (*RXR-γ*) rs10918169, *apolipoprotein A2* (*APOA2*) rs6413453, *calsequestrin 1* (*CASQ1*) rs617698 and *dual specificity phosphatase 12* (*DUSP12*) rs1503814 are screened as the susceptible genes and loci of T2DM belonging to this region [23]. Shi et al.’s study indicated that the allele frequencies of rs4240711, rs4842194 and rs3132291 variants in *RXR-α* gene were statistically different between metabolic syndrome (MetS) and normal controls in a Chinese Han population, and the genotypes of studied variants were associated with the decreased risk of MetS [24]. A collection of studies showed that the variants of *RXR-α* and *RXR-γ* genes were associated with the risk of T2DM [25–27].

GDM may have a similar genetic basis to T2DM and are considered to be an early stage of T2DM [28]. We propose the hypothesis that the functional genetic variations of *RXR-α/γ* genes may affect transcription or post-transcriptional regulation of genes and change the susceptibility of pregnant women to GDM. Hence, we investigated the relationship between the functional variations of candidate *RXR-α*/*γ* genes, and the risk of GDM in pregnancy women in Guangxi area by using a molecular epidemiological case–control study.

## Materials and methods

### Study subjects

All 1313 subjects were recruited from the Affiliated Hospital of Guilin Medical University, from September 2014 to June 2016. The project has been approved by the ethics committee of Guilin Medical University, and all subjects signed informed consent. All pregnant women were routinely offered a 75-g oral glucose tolerance test (OGTT) at 24–28 weeks and were evaluated according to the International Association of the Diabetes and Pregnancy Study Groups criteria (IADPSG). The GDM cases were identified when one or more values of glucose concentration were above cut-off points (≥5.10, ≥10.0 and ≥8.5 mmol/l at 0, 60 and 120 min, respectively). If a pregnant woman who had been previously diagnosed with endocrine diseases, serious systemic diseases, history of pre-pregnancy type 1 or type 2 diabetes and other pregnancy complications or long-term using of glucose metabolism affecting drugs, was excluded. In the present study, 573 GDM cases and 740 pregnant women with normal glucose tolerance at the same period were included.

### Research methods

#### Clinical and biochemical data

According to the study purpose, clinical indicators included age, height, weight, systolic blood pressure (SBP) and diastolic blood pressure (DBP) were collected. Meanwhile, body mass index before gestation (pre-BMI) was calculated as body weight (kg) divided by the square of height (m^2^). Five milliliter of peripheral venous blood from each subject was collected for detection of biochemical indicators. Biochemical data consisted of results of OGTT and glycosylated hemoglobin A1c (HbA1c).

#### Genomic DNA extraction

The genomic DNA was extracted from EDTA-treated whole blood using a DNA extraction kit (Aidlab Biotechnologies Co., Ltd, China) and stored in a refrigerator at −20°C prior to polymerase chain reaction (PCR).

#### SNP selection

The potential functional SNPs of candidate *RXR-α* and *RXR-γ* genes were screened by using NCBI dbSNP database (http://www.ncbi.nlm.nih.gov/projects/SNP), SNPinfo Web Server (http://snpinfo.niehs.nih.gov/) and SHEsis online tool (http://analysis.bio-x.cn/) [29], and should meet the following criteria: (1) SNPs with minor allele frequency (MAF) reported in HapMap based Han Chinese in Beijing (CHB) was greater than 0.05; (2) located at the regulatory region of studied genes (i.e., the 5′ near gene, 5′ untranslated regions [UTRs], 3′ UTR, 3′ near gene or splice sites); (3) might affect the microRNA–mRNA binding sites (MMBSs) activity, transcription factor-binding sites (TFBSs) activity in the putative promoter region or the post-transcriptional splicing process; (4) the linkage disequilibrium (LD) coefficient D′ or r^2^ < 0.8 between candidate SNPs.

According to the selection strategy, three SNPs including rs4842194 G>A in the *RXR-α* gene, and rs100537 A>G and rs2134095 T>C in the *RXR-γ* gene were identified for further study. Among them, rs4842194 G>A located at the 3′-UTR region of *RXR-α* gene may affect the MMBS activity. The SNP rs100537 A>G located in the 5′ near gene may affect the TFBS function, and rs2134095 T>C in the splice sites may affect the post-transcriptional splicing process of *RXR-γ* gene.

#### Genotyping

The PCR and restriction fragment length polymorphism (RFLP) methods were used to analyze the genotypes of the studied loci. The specific primers were designed and synthesized according to the sequence of candidate SNPs by Suzhou Hongxun Biotechnology Co., Ltd, China, in Table 1.

**Table 1:** The information of primer sequences for selected SNPs

Primer name	Primer sequence	Amplified length	Restriction enzyme
rs4842194-F	5′-*CCGTCCTGAGGCAGAGTGTTG*-3′	299 bp	*Pst I*
rs4842194-R	5′-*TCCCAAGAAGCAGCCGACC*-3′		
rs100537-F	5′-*CCTGGCACGGTGACTTGA*-3′	667 bp	*Msp I*
rs100537-R	5′-*TGGGAAATACGAATGACTGGAT*-3′		
rs2134095-F	5′-*CTTGCTGTGCCTGTTG*-3′	525 bp	*Hinf I*
rs2134095-R	5′-*GTGGAAGAGGGCTGAA*-3′		

Abbreviations: F, the forward primer; R, the reverse primer.

Each 20-μl PCR mixture contained genomic DNA 1 μl (100–200 ng), 2× M_5_ Taq HiFi PCR mix 10 μl, primer F (5 μM) 1 μl, primer R (5 μM) 1 μl and ddH_2_O 7 μl. Reaction procedures were as follows: pre-degenerated at 94°C for 4 min, denatured at 94°C for 30 s, annealed at 56–60°C for 25 s, extended at 72°C for 25 s, followed by 30 cycles and with a final extension at 72°C for 7 min. The restriction enzyme digestion gave rise to RFLP of the PCR products. Each digestion reaction mixture with PCR products 8 μl, FastDigest Green Buffer (10×) 1 μl, restriction enzyme 1 unite and ddH_2_O 6 μl was incubated in water at 37°C for 2 h, and then separated by 2.0% agarose gel electrophoresis.

#### Statistical analysis

The chi-square (χ^2^) test was used to assess the differences of selected demographic data, environmental exposure factors and genotypes of studied variants between cases and controls. A χ^2^ goodness-of-fit test was performed to determine whether the distribution about genotypes of SNPs in control group conformed to Hardy–Weinberg equilibrium (HWE). Unconditional logistic regression analysis was used to estimate the associations between the genotypes of SNPs and the risk of GDM by computing the odds ratios (ORs) and their 95% confidence intervals (CIs). In the present study, the two-sided test was used, and *P*≤0.05 was considered statistically significant. Bonferroni’s correction of the significance level (0.05/3 = 0.017) according to the number of comparisons was applied to account for multiple testing. The statistical analysis was performed with SPSS 17.0 (SPSS, Chicago, IL, U.S.A.). In addition, false positive report probability (FPRP) was estimated to assess the robustness of findings statistically significant association by using the method described by Wacholder et al. [30]. The FPRP threshold was set to 0.2, and the prior probability was set to 0.1 to detect the noteworthiness for OR of 1.5 or 0.67, with an α level equal to the observed *P*-value.

The multiple dimension reduction (MDR) software (version 3.0.2) was used to investigate the joint effect of SNPs. A 10-fold cross-validation and 1000-fold permutation testing were adopted under the null hypothesis of no association. The cross-validation consistency (CVC) and the testing balanced accuracy was help to identify the best model among all possibilities considered [31,32].

#### Functional prediction of studied variants

The online bioinformatics tool SNPinfo Web Server (https://snpinfo.niehs.nih.gov/cgi-bin/snpinfo/snpfunc.cgi) and Human Splicing Finder 3.1 [33] were conducted to investigate the putative functional effect of the variants and the gene alternative splicing regulatory.

## Results

### Characteristics of research objects

In the study, 1313 pregnant women in Guilin were recruited, including 573 patients in the GDM case group, with an average age of 31.48 ± 4.74 years; 740 patients in the normal pregnancy group, with an average age of 28.91 ± 4.18 years. The comparison of general data showed that age, pre-pregnancy BMI, SBP, DBP, 0/60/120 min OGTT and HbA1c in the cases were much higher than that of controls (*P*<0.05) as shown in Table 2.

**Table 2:** Demographic and clinical characteristics in GDM cases and controls (x ± s)

Variables	Cases (*n*= 573)	Controls (*n*=740)	*t*	*P*-values
Age (years)	31.48 ± 4.74	28.91 ± 4.18	10.25	<0.001
Pre-pregnancy BMI (kg/m^2^)	23.14 ± 3.63	21.66 ± 3.06	7.83	<0.001
SBP (mmHg)	111.52 ± 10.60	109.28 ± 9.48	3.98	<0.001
DBP (mmHg)	71.42 ± 7.88	69.96 ± 6.73	4.37	<0.001
OGTT 0 min glucose (mmol/l)	5.23 ± 1.32	4.41 ± 0.36	14.36	<0.001
OGTT 60 min glucose (mmol/l)	9.76 ± 2.23	7.00 ± 1.43	25.80	<0.001
OGTT 120 min glucose (mmol/l)	8.31 ± 2.15	6.10 ± 1.10	22.39	<0.001
HbA1c (%)	5.44 ± 0.68	4.93 ± 0.70	13.17	<0.001
Triglyceride (mmol/l)	2.67 ± 1.20	2.37 ± 1.03	4.90	<0.001
Total cholesterol (mmol/l)	5.37 ± 1.16	5.24 ± 1.07	2.18	0.029
HDL-c (mmol/l)	1.61 ± 0.37	1.66 ± 0.41	2.09	0.036
LDL-c (mmol/l)	3.49 ± 1.03	3.35 ± 1.04	2.29	0.022

Abbreviations: HDL-c, high -ensity lipoprotein cholesterol; LDL-c, low-density lipoprotein cholesterol.

### Relationship between studied variants and GDM risk

The genotypes of candidate SNPs were detected by a PCR-RFLP method. The results showed that the genotype frequencies of rs4842194 G>A, rs100537 A>G and rs2134095 T>C were in agreement with HWE in the controls (*P*>0.05), and the *P*-values were 0.727, 0.668 and 0.418, respectively. The D′ and r^2^ values were calculated to evaluate the LD of studied SNPs from their genotyping data by using the SHEsis online tool, and showed that there was no significant degree of LD detected among three SNPs (data not shown). A multivariable logistic regression analysis adjusted for age and BMI showed that the rs2134095 T>C in *RXR-γ* gene was significantly associated with the risk of GDM, individuals with CT genotype were 1.32-times higher than those with TT genotype (adjusted OR = 1.32, 95% CI = 1.01–1.73, *P*=0.043). However, the significance did not exist after Bonferroni’s correction. Further, under recessive genetic model, the CC genotype of rs2134095 could significantly reduce the risk of GDM compared with TT/CT genotypes (adjusted OR = 0.71, 95% CI = 0.56–0.90, *P*=0.004). We failed to find a significant association between the *RXR-α* rs4842194 G>A and *RXR-γ* rs100537 A>G and susceptibility to GDM in present study (*P*>0.05) as shown in Table 3.

**Table 3:** Association between genotypes of selected SNPs and GDM susceptibility

Gene	Genotype	Case	Control	*P* ^1^	Crude OR (95% CI)	*P* ^2^	Adjusted OR (95% CI)	*P* ^3^
*RXR-α*	rs4842194							
	GG	235	327	0.326	1		1	
	GA	263	333		1.10 (0.87–1.39)	0.427	1.05 (0.82–1.35)	0.68
	AA	75	80		1.31 (0.91–1.86)	0.144	1.31 (0.90–1.91)	0.164
	AA/GA	338	413	0.249	1.14 (0.91–1.42)	0.249	1.10 (0.87–1.39)	0.418
	GG/GA	498	660	0.227	1		1	
	AA	75	80		1.24 (0.89–1.74)	0.205	1.27 (0.89–1.82)	0.183
*RXR-γ*	rs100537							
	AA	48	55	0.199	1		1	
	AG	257	302		0.98 (0.64–1.49)	0.907	0.87 (0.56–1.36)	0.546
	GG	268	383		0.80 (0.53–1.22)	0.3	0.78 (0.50–1.21)	0.268
	GG/AG	525	685	0.536	0.88 (0.59–1.32)	0.528	0.82 (0.54–1.26)	0.364
	AA/AG	305	357	0.075	1			
	GG	268	383		0.82 (0.66–1.02)	0.073	0.88 (0.70–1.10)	0.259
	rs2134095							
	TT	158	219	**0.004**	1		1	
	TC	324	357		1.26 (0.98–1.62)	0.076	1.32 (1.01–1.73)	0.043
	CC	91	164		0.77 (0.55–1.07)	0.117	0.85 (0.60–1.20)	0.363
	CC/TC	415	521	0.425	1.10 (0.87–1.41)	0.422	1.17 (0.91–1.52)	0.221
	TT/TC	482	576	**0.003**	1			
	CC	91	164		**0.72 (0.58**–**0.89)**	**0.003**	**0.71 (0.56**–**0.90)**	**0.004**

The significance level after Bonferroni's correction for multiple testing is 0.017. Bold values indicate that the difference is statistically significant.

1 Two-sided χ^2^ test for genotypes’ distributions between cases and controls.

2 Unconditional logistic regression analysis.

3 Adjusted for age, pre-BMI in logistic regression models.

We also estimated the rs2134095 T>C on GDM risk with a stratified analysis under the recessive genetic model. The results showed that compared with TT/CT genotypes, the rs2134095 CC genotype could significantly reduce the risk of GDM in age > 30 years old subjects (adjusted OR = 0.61, 95% CI = 0.39–0.97, *P*=0.037) and in BMI > 22 kg/m^2^ subgroup (adjusted OR = 0.46, 95% CI = 0.30–0.70, *P*<0.001), and increase the GDM risk in SBP > 120 mmHg people (adjusted OR = 1.96, 95% CI = 1.14–3.36, *P*<0.015), DBP ≤ 80 mmHg people (adjusted OR = 1.33, 95% CI = 1.04–1.71, *P*<0.023), DBP > 80 mmHg people (adjusted OR = 2.33, 95% CI = 1.15–4.75, *P*<0.020), HbA1c < 6.5% subgroup (adjusted OR = 1.41, 95% CI = 1.11–1.78, *P*<0.004), TG ≤ 1.7 mmol/l (adjusted OR = 2.57, 95% CI = 1.45–4.53, *P*<0.001), TC ≤ 5.18 mmol/l subgroup (adjusted OR = 1.58, 95% CI = 1.13–2.22, *P*<0.007), high-density lipoprotein cholesterol (HDL-c) ≤ 1.5 mmol/l subjects (adjusted OR = 1.70, 95% CI = 1.16–2.49, *P*<0.006) and LDL-c > 3.12 mmol/l subjects (adjusted OR = 1.47, 95% CI = 1.08–2.00, *P*<0.013). We also found that rs2134095 interacted with age (*P_interaction_*=0.039), pre-BMI (*P_interaction_*=0.040) and TG (*P_interaction_*=0.025) influencing individual’s susceptibility to GDM in the recessive genetic model as shown in Table 4.

**Table 4:** Stratification analysis for associations between *RXR-γ* rs2134095 T>C and GDM risk

Variables	TT/TC (case/control)	CC (case/ control)	Crude OR (95%CI)	*P^1^*	Adjusted OR (95% CI)	*P^2^*	*P^3^*
Age (years)							**0.039**
≤30	210/395	49/117	0.79 (0.54–1.14)	0.210	0.78 (0.53–1.14)	0.202	
>30	272/181	42/47	**0.60 (0.38**–**0.94)**	**0.026**	**0.61 (0.39**–**0.97)**	**0.037**	
Pre-BMI (kg/m^2^)							**0.040**
≤22	187/361	48/90	1.03 (0.70–1.52)	0.884	1.06 (0.701.59)	0.787	
>22	295/215	43/74	**0.42 (0.28–0.64)**	**<0.001**	**0.46 (0.30–0.70)**	**<0.001**	
SBP (mmHg)							
≤120	206/324	243/299	**1.28 (1.00–1.63)**	**0.048**	1.29 (1.00–1.67)	0.055	0.165
>120	43/59	81/58	**1.92 (1.14–3.22)**	**0.014**	**1.96 (1.14–3.36)**	**0.015**	
DBP (mmHg)							0.134
≤80	214/347	271/333	**1.32 (1.04–1.67)**	**0.020**	**1.33 (1.04–1.71)**	**0.023**	
>80	35/36	53/24	**2.27 (1.16–4.44)**	**0.016**	**2.33 (1.15–4.75)**	**0.020**	
HbA1c (%)							
≤6.5	238/382	309/357	**1.39 (1.12–1.74)**	**0.004**	**1.41 (1.11–1.78)**	**0.004**	**-**
>6.5	11/1	15/0	**-**	**-**	**-**	**-**	
TG (mmol/L)							
≤1.7	30/106	55/81	**2.40 (1.41–4.08)**	**0.001**	**2.57 (1.45–4.53)**	**0.001**	**0.025**
>1.7	219/277	269/276	1.23 (0.97–1.57)	0.093	1.22 (0.94–1.58)	0.133	
TC (mmol/l)							0.761
≤5.18	118/208	142/175	**1.43 (1.04–1.96)**	**0.027**	**1.58 (1.13–2.22)**	**0.007**	
>5.18	131/175	182/182	1.34 (0.98–1.81)	0.063	1.21 (0.87–1.67)	0.256	
HDL-c (mmol/l)							0.554
≤1.5	96/147	128/129	**1.52 (1.07–2.17)**	**0.021**	**1.70 (1.16–2.49)**	**0.006**	
>1.5	153/236	196/228	**1.33 (1.00–1.75)**	**0.047**	1.27 (0.95–1.70)	0.112	
LDL-c (mmol/l)							0.670
≤3.12	105/168	125/152	1.32 (0.94–1.85)	0.113	1.29 (0.90–1.85)	0.160	
>3.12	144/215	199/205	**1.45 (1.09–1.93)**	**0.011**	**1.47 (1.08–2.00)**	**0.013**	

1 Unconditional logistic regression analysis.

2 Adjusted for age, pre-BMI in logistic regression models.

3 Test for multiplicative interaction obtained from logistic regression models.

### FPRP analysis

The FPRP test was adopted to assess the noteworthiness of the observed significant associations between the studied rs2134095 T>C SNP and the risk of GDM, and the prior probability of 0.1 and a relatively stringent FPRP cut-off value of 0.2 were set. The FPRP values were 0.050, 0.031, 0.049, 0.149, 0.176 and 0.178 for the associations of rs2134095 and GDM risk in all subjects, and subgroups of BMI > 22 kg/m^2^, HbA1c ≤ 6.5%, TC ≤ 5.18 mmol/l, HDL-c ≤ 1.5 mmol/l and LDL-c > 3.12 mmol/l, respectively, under the recessive genetic model (CC vs. TT/TC). It suggested that the above associations were noteworthy findings as presented in the present study as shown in Table 5.

**Table 5:** FPRP analysis for the significant associations of the rs2134095 T>C and GDM risk

Comparisons	Adjusted OR (95% CI)	Prior probability					
		0.25	0.1	0.01	0.001	0.0001	0.00001
TC vs. TT	1.32 (1.01–1.73)	0.136	0.321	0.839	0.981	0.998	1.000
CC vs. TT/TC	0.71 (0.56–0.90)	0.017	**0.050**	0.367	0.854	0.983	0.998
Subgroup							
Age > 30 years	0.61 (0.39–0.97)	0.238	0.484	0.912	0.990	0.999	1.000
BMI > 22 kg/m^2^	0.46 (0.30–0.70)	0.010	**0.031**	0.259	0.779	0.972	0.997
SBP > 120 mmHg	1.96 (1.14–3.36)	0.210	0.444	0.898	0.989	0.999	1.000
DBP (mmHg)							
≤80	1.33 (1.04–1.71)	0.078	0.203	0.737	0.966	0.966	1.000
>80	2.33 (1.15–4.75)	0.347	0.614	0.946	0.994	0.999	1.000
HbA1c ≤ 6.5%	1.41 (1.11–1.78)	0.017	**0.049**	0.360	0.850	0.983	0.998
TG ≤ 1.7 mmol/l	2.57 (1.45–4.53)	0.092	0.234	0.771	0.971	0.997	1.000
TC ≤ 5.18 mmol/l	1.58 (1.13–2.22)	0.055	**0.149**	0.658	0.951	0.995	0.999
HDL-c ≤ 1.5 mmol/l	1.70 (1.16–2.49)	0.066	**0.176**	0.701	0.960	0.996	1.000
LDL-c > 3.12 mmol/l	1.47 (1.08–2.00)	0.067	**0.178**	0.705	0.960	0.996	1.000

Bold values indicate that the difference is statistically significant at the test level of α=0.2.

### High-order interaction with GDM risk by MDR analysis

The results of MDR analysis indicated that rs2134095 was the best one-factor model for GDM with a maximum CVC of 10/10, a testing balanced accuracy of 0.531, and a *P*-value of statistical test of 0.003. More interestingly, the best interaction model to predict GDM risk for the present study population was a two-factor model of rs2134095 and rs100537 with a maximum CVC of 10/10, a testing balanced accuracy of 0.552, and a *P*-value <0.001 (Table 6).

**Table 6:** MDR analysis for the GDM risk prediction

Best model	Training balanced accuracy	Testing balanced accuracy	CVC	*λ*²	*P*
1	0.542	0.531	10/10	8.91	**0.003**
1, 2	0.555	0.552	10/10	17.62	**<0.001**
1, 2, 3	0.574	0.540	10/10	27.04	**<0.001**

Labels: 1. rs2134095; 2. rs100537; 3. rs4842194.

*P*-value for 1000-fold permutation test.

The best model was selected as the one in boldface with the maximum prediction precision and CVC.

### Functional prediction of rs2134095

**Figure 1:**
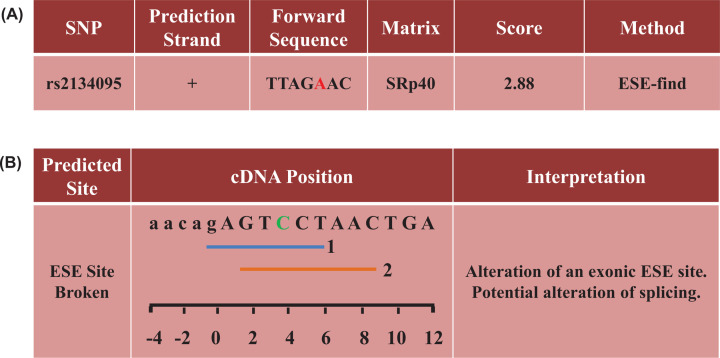
Predicted the function of rs2134095 on *RXR-γ* post-transcriptional splicing sites (**A**) Predicted by SNPinfo Web Server (base complementary pairing principle: A=T, G≡C). (**B**) Predicted by Human Splicing Finder 3.1 tool.

Based on the positive findings identified above, the *RXR-γ* rs2134095 was further explored of a putative alteration in regulation of gene splicing. It seems that the variant has an impact in splicing regulation according to SNP Function Prediction of SNPinfo Web Server and Human Splicing Finder 3.1 tool. The variant is located in an acceptor splicing site, and alteration of the C→T allele of rs2134095 leads to the disruption of an exonic splicing enhancer (ESE) SRp40 (Figure 1). It is biologically reasonable to hypothesize that the variant has an effect on the splicing of the *RXR-γ* mRNA, thus altering the function of the gene product.

## Discussion

GDM is characterized by β-cell dysfunction, insulin secretory defect and peripheral insulin resistance. It is likely to bring adverse consequences for both mothers and their offspring, for instance, pregnant women with GDM are often at a relatively higher risk of T2DM, obesity, dyslipidemia, preterm birth, spontaneous abortion, respiratory distress syndrome of newborn etc. Studies showed that the RXR-α/γ proteins perform the function of transcription factors by binding as heterodimers to the specific sequences in the promoter of target genes of VD and retinoid pathways, which are closely related to diabetes, islet function, and glucose and lipid metabolism. We believe that it is necessary to explore the relationship between RXR-α/γ and GDM. GWASs and meta-analysis have shown that association genetic loci could provide insight into pathogenetic mechanisms underlying the GDM [20,34–36]. To date, there have been studies on multiple genes and their SNPs of VD pathway with the susceptibility to T2DM and GDM [26,37–39]. Studies have shown that the variants in *RXR-α*/*γ* genes might be associated with the risk of T2DM and metabolic syndrome [24–26]. We genotyped three potential functional SNPs in *RXR-α*/*γ* genes explore the pathogenesis of GDM.

In the single locus analysis, we found that the *RXR-γ* rs2134095 T>C was significantly associated with the risk of GDM under the recessive genetic model in a Southern Chinese population. In the stratified analysis, the reduced risk of GDM associated with rs2134095 SNP was more pronounced among the elderly (>30 years old) and relatively higher pre-BMI (>22 kg/m^2^) subjects carrying the CC genotype in the recessive genetic model. Further, the interaction between rs2134095 and age and pre-BMI was detected. The above findings suggest that not only a single environmental or genetic risk factor but also their interaction might affect the individual suffering from GDM under different etiologies. Additionally, recent studies have confirmed that the variants located in *RXR-γ* were significantly associated with the lipid metabolism of chronic metabolic diseases. These variants are closely related to abnormal lipid metabolism by affecting the levels of TG, LDL-c, apolipoprotein B etc [40–42]. In the hierarchical analysis of this study, higher risk effects in HbA1c (≤6.5%), TG (≤1.7 mmol/l), TC (≤5.18 mmol/l) and LDL-c (>3.12 mmol/l) are observed in rs2134095CC carrier subjects. These findings might be helpful to clarify the genetic component of *RXR-γ* and lipid profile.

Very interesting, results from the MDR analysis also consistently identified *RXR-γ* rs2134095 T>C as the main single susceptibility locus contributed to the risk of GDM, and some complex gene–gene interaction effects exist among the *RXR-γ* SNPs. It showed that a two-factor model including the combination of rs2134095 and rs100537, is the best model to predict GDM risk in the study population. These results suggest that *RXR-γ* gene variants may have a joint effect on the genesis of GDM.

These above findings seem to be biologically plausible. Study confirmed that gene variants might change the splicing pattern of pre-mRNA leading to a wrong transcript product and ultimately influence the gene expression or function [43–45]. According to the online prediction database ‘snpinfo web server’, the SNP rs2134095 may play the role of exonic splicing enhancer (ESE) or silencer (ESS) performing a function of *RXR-γ* gene post transcriptional splicing. Grave et al. used a general linear model to evaluate the VD pathway gene–gene interaction and detected a significant effect of the interaction between *RXR-γ* rs2134095 and GC rs7041 on low-density lipoprotein cholesterol (LDL-c) levels. The further functional analysis indicated that the studied variants were involved in the regulation of the gene expression [42]. Another study of Sentinelli et al*.* found that the ‘at-risk’ haplotype of *RXR-γ* gene variants (rs1128977, rs2651860, rs2134095, rs283696 and rs10918169) were associated with the risk of familial combined hyperlipidemia (FCHL) and a higher LDL-c level [40]. It is speculated that the rs2134095 T>C may affect the function of RXR-γ and alter the activation of retinoid and VD pathway genes, which are closely related to diabetes, islet function, and glucose and lipid metabolism. In addition, maternal plasma levels of vitamin A and vitamin D reduce during pregnancy, thus exacerbating the functional impairment of above pathways caused by rs2134095. The above is likely to explain the mechanism of the changes in the susceptibility of pregnant individual to GDM.

The FPRP analysis is an effective method to verify the noteworthiness of significant associations [46]. In the present study, a relatively stringent FPRP threshold of 0.2 was set. The FPRP values of the observed significant associations between rs2134095 T>C and the risk GDM is much lower than the preset threshold 0.2 in BMI > 22 kg/m^2^, HbA1c ≤ 6.5%, TC ≤ 5.18 mmol/l, HDL-c ≤ 1.5 mmol/l and LDL-c > 3.12 mmol/l subgroups under the recessive genetic model. It suggests that the positive findings are probability authentic and reliable. Meanwhile, the detected FPRP values in some comparisons much greater than 0.2 indicated that these significant findings might be chance observations. Therefore, the conclusion obtained here must be viewed as preliminary and needs to be verified.

To our knowledge, it was the first time to explore the relationship between the studied functional variants of *RXR-α*/*γ* genes and the risk of GDM, and some etiological clues of GDM are suggested from the perspective of genetics. The strengths of our study include its good design, relatively large sample size and multiple analysis methods. However, some limitations in this study should not be neglected. First, designed as a hospital-based case–control study, it may be still subject to inherent biases, like selection bias and recall bias. Second, although relatively large samples were recruited, however, the lower minor allele frequency of some tested SNPs might still limit the statistical power to detect significant association in some subgroups. Third, some positive correlations between rs2134095 T>C and GDM risk were observed, however, the specific mechanisms underlying the effect of the above-mentioned positive findings should be investigated.

## Conclusion

In summary, the present study support that the *RXR-γ* gene rs2134095 T>C variant is significant associated with the increased risk of GDM by effect of a single locus and/or complex joint gene–gene and gene–environment interactions. Furthermore, larger sample-size and different population studies are required to confirm our findings.

## Data Availability

The data used to support the findings of the present study are included within the article.

## Abbreviations

BMI: body mass index
CI: confidence interval
CVC: cross-validation consistency
ESE: exonic splicing enhancer
FPRP: false positive report probability
GDM: gestational diabetes mellitus
GWAS: genome-wide association study
HbA1c: glycosylated hemoglobin A1c
HDL-c: high-density lipoprotein cholesterol
HWE: Hardy–Weinberg equilibrium
LD: linkage disequilibrium
LDL-c: low-density lipoprotein cholesterol
MDR: multiple dimension reduction
MMBS: microRNA–mRNA binding site
OGTT: oral glucose tolerance test
OR: odds ratio
PCR: polymerase chain reaction
RFLP: restriction fragment length polymorphism
RXR*α/γ*: *retinoid X receptor* *-α/γ*
SBP: systolic blood pressure
SNP: single nucleotide polymorphism
TC: total cholesterol
TFBS: transcription factor-binding site
TG: triglyceride
T2DM: type 2 diabetes
UTR: untranslated region
VD: vitamin D
